# Modelling changing patterns in the COVID‐19 geographical distribution: Madrid’s case

**DOI:** 10.1111/1745-5871.12521

**Published:** 2021-11-09

**Authors:** Adolfo Maza, María Hierro

**Affiliations:** ^1^ Department of Economics University of Cantabria Santander Spain

**Keywords:** changing patterns, COVID‐19, geographical factor, lockdown, Madrid, spatial ecological model

## Abstract

We analyse the transmission factors shaping the spatial distribution of COVID‐19 infections during the distinct phases of the pandemic’s first wave in Madrid, Spain, by fitting a spatial regression model capturing neighbourhood effects between municipalities. Our findings highlight that factors such as population, mobility, and tourism were instrumental in the days before the national lockdown. As a result, already in the early part of the lockdown phase, a geographical pattern emerged in the spread of the disease, along with the positive (negative) impact of age (wealth) on virus transmission. Thereafter, spatial links between municipalities weakened, as the influences of mobility and tourism were eroded by mass quarantine. However, in the de‐escalation phase, mobility reappeared, reinforcing the geographical pattern, an issue that policymakers must pay heed to. Indeed, a counterfactual analysis shows that the number of infections without the lockdown would have been around 170% higher.

## Graphical abstract

Explanatory factors of the spread of the pandemic in Madrid changed according to the phase of national lockdown. As for the effectiveness of social distance measures, a counterfactual analysis shows that the number of infections without the lockdown would have been around 170% higher.

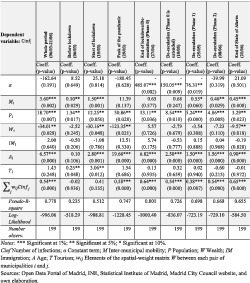

**Key insights**

Transmission factors shaping the spatial distribution of COVID‐19 infections during the distinct phases of the pandemic first wave in Madrid are examined via a spatial regression model capturing neighbourhood effects between municipalities. Findings highlight that explanatory factors, which have a geographical component linked to mobility, are dependent and thus change according to the phase of national lockdown. As for the effectiveness of social distance measures, a counterfactual analysis shows that the number of infections without the lockdown would have been around 170% higher.

## 1 INTRODUCTION

COVID‐19 arguably possesses peculiarities that make it a unique and significant shock. On one hand, it combines, probably like no other, both demand‐ and supply‐driven impacts. On the other hand, it poses a trade‐off dilemma between health and economic outcomes because mobility‐based restrictions aimed at flattening the incidence curve have led inevitably to reduced productivity. Its influence extends to other spheres of life, to the point that, as Burton (2021) has suggested, everyone nowadays is familiar with terms such as “social distancing,” “lockdown,” “flatten the curve,” “telework,” “risk group,” “herd immunity,” “cabin fever,” and so on.

Consequently, growing numbers of articles address the COVID‐19 pandemic from different perspectives, some of which have attempted to shape the unequal and changing spatial/geographical distribution of the COVID‐19 disease. To this end, academic efforts have focused on ecological studies to analyse the determinants of COVID‐19 infections and/or deaths.

Contributing to such efforts, in this article we endeavour to advance a better understanding of the determinants of COVID‐19 community transmission by comprehensively addressing critical points that should not be (but often have been) overlooked. First, due to the interactions between them, different potential drivers of COVID‐19 propagation have to be considered together and not individually as done in many papers; consequently, and bearing in mind data limitations, here we propose and estimate a model that tries to assess the role played by some of these drivers. Second, spatial interactions between neighbouring areas or, more technically, the existence of spatial correlation/dependence, should be included in the model; this issue is core because the geographical location of territories matters when it comes to explaining the spread of a virus. Our thinking aligns with that provided by Bissell (2021, p. 157): “my wager is that geography matters now more than ever as we try to move forward and refashion our lives in the long comet tail of COVID‐19.” Third, it is crucial to gauge whether and, depending on the phase of the pandemic, how driving factors vary over time hand‐in‐hand with the application or relaxation of non‐pharmaceutical interventions such as social/physical distancing.

To accomplish our aim, we made several important decisions, of which perhaps the most significant concerns the case study. We refer both to the country/region analysed and to the level of disaggregation of data used. We chose the Autonomous Community of Madrid (Spain) and used municipal level data; although Madrid consists of 179 municipalities, we took data for 178 of them and for the 21 districts that make up the municipality that bears the same name of the region (Madrid), which is disproportionately large to be considered as just one more.^1^ This is an interesting case study from which valuable lessons can be obtained, because Madrid represents the “epicentre” of the pandemic in Spain, one of the countries hardest hit by the virus (Arango, 2020; Paez et al., 2021; Trias‐Llimós & Bilal, 2020).

At this point, we focus on the first wave of the pandemic because it is, at least for the time being, the only wave curbed in Spain by a national lockdown (enacted on 15 March 2020). The period under study (6 March–21 June 2020) contains very different phases of the pandemic, especially in terms of non‐pharmaceutical intervention measures (no restrictions, lockdown, de‐escalation phases). Thus, the analysis is carried out for the whole period and for specific dates within it. That approach provides an accurate analysis of the evolution of the first wave of the pandemic and, more importantly, of potential changes in explanatory factors of virus transmission over time. This approach, in line with that used by Ehlert (2020), allows us to assess how the social stratification of COVID‐19 shifts between the different phases of the pandemic.

Keeping all these considerations in mind, the rest of the article is structured as follows. In Section 2, a brief review is presented of recent but extensive literature on the COVID‐19 pandemic. In Section 3, we propose a causal model to unveil factors underlying the geographical distribution of the pandemic based on theoretical foundations and empirical work, as well as data availability. Section 4 reports the main results when applied to our case study. Section 5 summarises the main conclusions of the paper.

## 2 LITERATURE REVIEW

Since the outbreak of COVID‐19, a vast amount of research based on clinical studies has been conducted to identify individual‐level risk factors for infection. Less prevalent have been ecological studies on the main social, economic, and demographic factors associated with geographical patterns of local transmission affecting the design of comprehensive and coordinated actions to combat the spread and unequal impact of the virus across territories. Clearly, “knowledge of these factors and the ways in which they are spread is crucial, since at the current state of research it seems not to be possible to contain the pandemic by medical means alone” (Ehlert, 2020, pp. 2–3). The literature is also very diverse in terms of the spatial framework, methodology employed, and evidence provided.

Starting with the spatial framework, in light of what has happened with the advance of the virus, we know that, despite its global character, it has different impacts across countries and territories within countries. Precisely because of that spatial heterogeneity (highlighted by Amdaoud et al., 2021), uncovering the fundamental drivers of virus dissemination has become a priority. Some examples of cross‐country analysis of the spread of COVID‐19 include works by Mogi and Spijker (2021) and Sannigrahi et al. (2020) for Europe and Jain and Singh (2020) and Hassan et al. (2020) at a global scale. Conversely, studies adopting a regional perspective on the socio‐economic dimension of the pandemic include, for instance, those by Ehlert (2020) for Germany, Buja et al. (2020) and Ascani et al. (2020) for Italy, Ramírez‐Aldana et al. (2020) for Iran, and Orea and Álvarez (2020) for Spain. Finally, there are papers dealing with the spread of COVID‐19 at a municipal/district level, such as that by Plümper and Neumayer (2020) for German local districts, Boterman (2020) for the Netherlands, and Andersen et al. (2021) for United States’ counties. Others focus attention on specific cities such as in work by Baena‐Díez et al. (2020) for districts in Barcelona, Almagro and Orane‐Hutchinson (2020) and Hamidi and Hamidi (2021) for neighbourhoods in New York City, Vaz (2021) for neighbourhoods in Toronto, Pan et al. (2021) at the borough level in London, Yip et al. (2021) with census data for Hong Kong, and Qiu et al. (2020) for a sample of Chinese cities.

As for methodological considerations, traditional statistical techniques ranging from a straightforward correlation‐based method to cluster and factor analysis to linear and multinomial regression approaches have been widely used to mitigate the lack of knowledge on the determinants of COVID‐19. However, most such studies ignore the spatial interdependencies between the geographical areas under scrutiny and fail to capture “disease transmission pathways and network effects [and to quantify] the magnitude of spatial spillovers” (Krisztin et al., 2020, p. 210). To fill this gap are several ecological studies using relevant and different spatial econometric techniques (Andersen et al., 2021; Arenas et al., 2020; Bourdin et al., 2021; Cordes & Castro, 2020; Dehghan Shabani & Shahnazi, 2020; Guliyev, 2020; Krisztin et al., 2020; Mollalo et al., 2020; Orea & Álvarez, 2020; Ramírez‐Aldana et al., 2020; Sannigrahi et al., 2020; Xie et al., 2020). All confirm that the rates of COVID‐19 cases are spatially correlated.

In terms of empirical evidence, most COVID‐19 researchers have concluded that the virus disproportionally affects deprived communities and those with lower income levels, the elderly, and several ethnicities (Andersen et al., 2021; Berkowitz et al., 2020; Hassan et al., 2020; Pan et al., 2021; Plümper & Neumayer, 2020; Sannigrahi et al., 2020). Some authors postulate that heavily urbanised areas with high CO_2_ emissions, high average temperatures, and older demographic structures are more exposed to COVID‐19 risks (Hassan et al., 2020; Jain & Singh, 2020; Ramírez‐Aldana et al., 2020). Finally, other studies, such as that by Almagro and Orane‐Hutchinson (2020), have found that, in the early stages of the pandemic especially, some occupations channel the spread of the disease, and workers in jobs with a high degree of physical contact are more likely to contract the virus.

However, puzzling empirical findings remain to be explored. One set concerns the distinction between population density and population. Although counterintuitive, most studies conclude that infections do not seem to be significantly correlated with population density but rather with population levels (Boterman, 2020; Hamidi & Hamidi, 2021); according to Hassan et al. (2020), ways of transmission are multiple even in low(er) density areas at social events such as funerals or family gatherings. Nevertheless, some studies point to a positive and significant relationship between population density and COVID‐19 exposure (Buja et al., 2020; Mogi & Spijker, 2021). More also needs to be done in terms of the role of public transport in the spread of the disease, and no solid evidence exists that the use of public transport contributes to the spread of the COVID‐19 pandemic (Almagro & Orane‐Hutchinson, 2020; Hamidi & Hamidi, 2021).

## 3 THEORETICAL BACKGROUND AND PROPOSED MODEL

### 3.1 A standard model

When specifying a reference model for what drives the spatial transmission of COVID‐19, the first major decision concerned its dependent variable. We considered the number of infections registered in each municipality in the 14 days prior to the date analysed with data collected from the Open Data Portal of the Community of Madrid.^2^ The rationale is that daily data are too volatile to support ultimate conclusions. Put differently, by using a variable covering a 14‐day period, we can control for time‐variant heterogeneity to draw reliable findings. Even if the problem of volatility is overcome, the number of cases recorded in the early days of the pandemic is an estimate that is likely highly biased downwards because tests were performed only on hospitalised people. For this reason, to assess the spread of the virus, some studies use the number of excess deaths instead of the number of cases (Bartoszek et al., 2020). We preferred to use official data on the number of infections because, on one hand, discrepancies between sources regarding excess deaths were, at least in Spain and thus Madrid, quite remarkable, and on the other hand, reliability of excess deaths data at the municipal level is difficult to guarantee.

Regarding independent variables, for the sake of simplicity, we only focused our analysis on the so‐called COVID‐19 Determinants of Health Model, which represents an adaptation of the Kaiser Family Foundation Model (Kaiser Family Foundation, 2020) postulated by the Public Health Outcomes and Effects of the Built Environment (PHOEBE) Laboratory (University of Maryland, Phoebe Laboratory, 2020). This model theorises the attribution of individual and social determinants to COVID‐19 health outcomes, and therefore, its empirical application, explained below, allowed us to understand the heterogeneous and changing spatial/geographic distribution of the pandemic.

The Kaiser Family Foundation Model divides the determinants of health into six categories: economic environment, built environment, education environment, food environment, social environment, and health care environment.^3^ It is a general model designed to study the spread of COVID‐19 between countries but can be adapted and thus employed at lower geographical levels; we mean from states/regions down to, for instance, neighbourhoods/households. Thus, when analysing the propagation of the pandemic within a region, as in our case, some existing categories must be ruled out because differences between units of analysis, if any, are negligible (see Hu et al., 2021, who have proposed a model gauging the relationship between various social factors such as housing quality, living conditions, travel pattern, race/ethnicity, and income).

For our case study, three categories included in the general model were removed—education, food, and health care environments—because all municipalities have comparable conditions in relation to them. As for the three remaining categories, and constrained by data availability, we chose the following explanatory variables:

1. Inter‐municipality mobility ( M), which could be included in the built environment category. It is constructed by handling big data coming from *Studies on Mobility based on Mobile Phone* published by the Spanish National Statistical Institute (INE). The data source provides, on a daily basis, information on the percentage of population moving from each to another municipality of Madrid; although we coped with some significant problems in the computation of these data, which are explained in depth in the Data S1, we reckon this variable is crucial for our analysis. Hence, having overcome these problems and in line with the dependent variable, we computed, for the whole period and each of the dates mentioned below, a variable capturing average inter‐municipal mobility during the previous 14 days (we tried with 21 days, being the results roughly the same). In line with theoretical approaches assuming a link between mobility and disease transmission among citizens (see Kissler et al., 2020), our hypothesis (H1) is that the higher the mobility, the higher the number of infections.
2. Population ( P), which could be included as a demographic structure factor in the category of social environment. It is defined as the number of citizens (in logs), with data mainly taken (as in the remaining cases hereafter) from the Statistical Institute of the region of Madrid, apart from those for the districts of the municipality of Madrid, which are collected from the Madrid City Council website. The importance of this variable is beyond doubt because it is capturing “size.” It is instrumental in an ecological model trying to explain infections as ours and also in models devoted to many other issues. By way of example, traditional gravity models of migration (see Poot et al., 2016) assume that migratory flows between two areas are directly linked to their size in terms of population. Following the same assumption, our hypothesis (H2) is that highly populated areas are more prone to suffer from the pandemic, so a positive sign is expected for the estimated coefficient.^4^
3. Wealth ( W) is an important economic environment factor. It is defined as the gross disposable income (in logs). By choosing this variable, we wanted to assess whether people living in specific municipalities where relatively low disposable incomes prevail are more exposed to contagion. Several studies have concluded that the spread of COVID‐19 is faster in poor areas (Baena‐Díez et al., 2020; Kim & Bostwick, 2020; Patel et al., 2020), because of lower levels of access to economic, educational, health, and social resources. In that regard, Almagro and Orane‐Hutchinson (2020) also point to the strong presence of occupations with a high degree of social exposure/physical contact in the most deprived areas. Our hypothesis (H3) is, then, a negative relationship between the severity of the pandemic and wealth.
4. Immigration ( IM) is a social environment factor related to issues such as inclusion or cohesion emphasised in the COVID‐19 Determinants of Health Model. It is defined as the stock of foreigners (in logs). Postulated by, among others, Berkowitz et al. (2020), Chih and Ojede (2020), Khalatbari‐Soltani et al. (2020), Kim and Bostwick (2020), and Niedzwiedz et al. (2020), the idea is that racially segregated areas are more prone to contagion. Therefore, as a proxy for it, we include an immigration variable (in line with Fasani & Mazza, 2020). The hypothesis (H4) is now that the higher the stock of foreigners, the higher the chance of registering a significant number of contagions.^5^
5. Age ( A) is another demographic factor included in the social environment category. It is defined as the mean age of the population (we do not take logs here for the sake of interpretation). In this respect, and although the influence of age is broadly accepted (Gondim & Machado, 2020), the expected sign is not straightforward. Recall that we are examining the first wave of the pandemic, in which the positive cases were primarily related to people showing symptoms and indeed attending hospitals (at that time, the capacity for mass testing and monitoring was considerably weak). Thus, we presume a direct relationship between the dependent variable and age is more likely. Supporting this forecast was the precarious situation of nursing homes, and residents were disproportionally affected by the pandemic because of a lack of personal protective equipment for staff. Hence, our hypothesis (H5) calls for a positive coefficient linked to the age variable.^6^
6. Tourism ( T), as another built environment factor related to transportation. It is proxied by the number of places in hotels and other tourist establishments (in logs). A positive link is expected between the intensity of the pandemic and tourism because, in a scenario of unrestricted international travel flows, areas receiving many tourists would be likely to suffer a sharp increase in COVID‐19 cases. Therefore, the hypothesis (H6) is that the stronger the weight of tourism, the higher the number of infections.

At this point, having defined the set of potential determinants of the number of COVID‐19 infections, and it being obvious that they do not “work” independently and that “the cumulative and aggregate force of these determinants” (Hu et al., 2021, p. 12) needs to be tested, we can propose our reference model. Such a non‐spatial model would be as follows:

(1) $${\text{Cinf}}_{i}=\alpha +\beta_{1}M_{i}+\beta_{2}P_{i}+\beta_{3}W_{i}+\beta_{4}{\mathrm{IM}}_{i}+\beta_{5}A_{i}+\beta_{6}T_{i}+\varepsilon_{i},$$

where Cinf refers to the number of infections, *i* denotes a municipality, *β* are the estimated coefficients, and all independent variables have been denoted above.

### 3.2 An extended spatial model

Equation 1 would provide an incomplete ecological model for COVID‐19 infection. When modelling the virus incidence of reported cases, there is an instrumental factor that should not be overlooked: the potential link between the total number of infections in a municipality and that in neighbouring municipalities. As noted by Kuebart and Stabler (2020, p. 482), “infectious diseases should be understood as socio‐spatial processes with complex geographies.” That is to say, there is little doubt we have to consider a spatial extension of the benchmark model.

The existence of spatial spillovers can indeed be considered a well‐established finding from both theoretical and empirical perspectives. In terms of theory, Chih and Ojede (2020, p. 6) have noted that the COVID‐19 pandemic “is expected to exhibit global spatial spillovers rather than being spatially independent.” From this perspective, moreover, we could borrow a central idea from theories of the New Economic Geography and Economic Growth that a key component of agglomeration forces is agglomeration itself, which makes agglomeration processes strongly cumulative (Baumont et al., 2001). This idea fits neatly with the expansion of a virus because, even if its starting spatial distribution was arbitrary, the outbreak of the virus would likely lead to the formation of a geographical cluster composed of the first infected zone and neighbouring areas.

From an empirical point of view, why is the existence of notable spatial spillovers, especially in its initial stage, so relevant? Bernasco (2010, p. 118) has provided one insight, inasmuch as “in the explanation of social phenomena, it generally holds that the smaller the unit of analysis, the more urgent is the need to consider the presence of spatial interaction.” Therefore, if spatial diffusion processes are expected to occur between countries, such will be the case, and much more so, between municipalities. Concisely, the idea behind spatial spillovers lies in the natural occurrence of hotspots that, when time passes, are spread to closer areas mainly due to population mobility.

In any event, instead of taking these matters for granted, before moving forward, we confirmed the presence of geographical clusters by computing some spatial dependence statistics (Moran’s I and Geary’s C statistics), as shown in Data S2. We concluded that municipalities cannot be considered isolated units when specifying the model. Hence, a spatial ecological model capturing interactions between units should be proposed (for a comprehensive reference dealing with the geographical distribution of diseases and the use of spatial methods to model it, see Lawson, 2006).

Accordingly, we extend the model (Equation 1) by considering, as an additional independent variable, the spatial lag of the dependent one ( ${\left[\text{WCinf}\right]}_{i}=\sum_{j}w_{\mathrm{ij}}{\text{Cinf}}_{j}$), being our last hypothesis (H7) that its coefficient is positive. As for the definition of this variable, $w_{\mathrm{ij}}$ denotes the elements of the distance/spatial‐weight matrix *W* between each pair of municipalities *i* and *j*. Specifically, spatial weights are defined as the (standardised) inverse of the square of the distance between the corresponding centroids; the reason we use a square matrix is to impose a heavy penalty on distance, which seems to be logical when dealing with a unit of study as small as the municipalities. In any case, our results do not depend significantly on the distance matrix, as we also tried with other versions—such as the contiguity matrix, the inverse of the distance, or matrices considering different cut‐offs—and the results were roughly similar to those reported below.^7^ Consequently, the proposed final spatial model we use in the next section reads as follows:

(2) $${\text{Cinf}}_{i}=\alpha +\beta_{1}M_{i}+\beta_{2}P_{i}+\beta_{3}W_{i}+\beta_{4}{\mathrm{IM}}_{i}+\beta_{5}A_{i}+\beta_{6}T_{i}+\beta_{7}\sum_{j}w_{\mathrm{ij}}{\text{Cinf}}_{j}+u_{i}.$$

### 3.3 Some key dates

We now apply the model to the whole period covering the first wave of the pandemic in Madrid (6 March–21 June 2020) and, more importantly, to specific dates. We aim to detect changes in the explanatory factors that occur in response to non‐pharmaceutical intervention measures to better understand the changing spatial and temporal patterns of COVID‐19 spread. These dates are reported in Table 1.

**TABLE 1:** Key dates

Descriptive name	Date	Reasons for choosing that date	Number of cases in the previous 14 days (Madrid region)
Before lockdown	06/03	In our view, it is pertinent to begin with the time without any restrictions. We select this date because it was the first day with more than one municipality with a cumulative incidence above 25 cases per 100,000 inhabitants (the threshold for a risk of contagion according to the European Centre for Disease Prevention and Control)	250
Start of lockdown	15/03	The point in which the national government decided to declare the state of alarm and thereby limit mobility to the maximum.	4261
Peak of the pandemic	29/03	It is not an arbitrary date within the confinement period, but that on which the cumulative incidence was higher during the first wave	21,901
End of lockdown/de‐escalation (Phase 0)^a^	27/04	By this time, the government had devised a coordinated plan to get ready for a kind of normalcy in which restrictions were progressively released in an orderly fashion. In Phase 0 of the de‐escalation after the lockdown, there was a slight relaxation of mobility restrictions (outdoor sports, daily one‐hour walks, the opening of stores)	8580
De‐escalation (Phase 0 is extended)	10/05	Due to non‐compliance with requirements, Phase 0 was extended for 2 weeks in the region of Madrid	3258
De‐escalation (Phase 1)	25/05	Other mobility restrictions were removed, such as the opening up of terraces in bars and restaurants and small and medium‐size shops with capacity limits	1834
De‐escalation (Phase 2)	08/06	Additional mobility restrictions were eliminated, especially concerning the restaurant sector	1483
End of state of alarm	21/06	Our study period ends with the moment when the national government declared the expiration of the (first) state of alarm. It implied the start of the “new normality”	1104

*Source*: Open Data Portal of Madrid and own elaboration.

^a^ Although the official date for the end of the lookdown was 4 May, we chose this earlier date because it is, in fact, the one from which some measures (called by the government “relief measures”) were taken to allow some mobility.

To summarise, we can highlight the different incidences of the virus in the Madrid region as a whole throughout the period analysed. In less than a month, Madrid went from 250 cases in the 14 days prior to 6 March to almost 22,000 on 29 March. From that date onwards, the incidence of the virus fell until just over 1000 cases were recorded on the date of the end of the state of alarm (21 June). Table 1 also shows that different non‐pharmaceutical intervention measures were implemented and withdrawn during that period, including the start of the state of alarm with lockdown and different de‐escalation phases. The finding makes our case study compelling because it offers the possibility of detecting changes in driving factors of the pandemic that are scenario dependent.

## 4 RESULTS

In this section, we present the results and extract the most relevant information from the various estimates of our proposed model (Equation 2). First, Table 2 shows some descriptive statistics—mean, maximum, minimum, and coefficient of variation—of the variables included in Equation 2; this is for the whole period to avoid repetition. The most significant feature is that inequality (last column) is much higher in the number of infections than in the rest of the variables. Although approximately 7,700 people were infected (always for a 14‐day period) on average in Madrid during the first wave of the pandemic, some municipalities were free of COVID‐19 (no cases were recorded) and some had an average of c.400 cases. This unequal incidence is what our model tries to explain.

**TABLE 2:** Data: Descriptive statistics for the whole period

*Variable*	Mean	Maximum	Minimum	Coefficient of variation
Number of infections ( Cinf)	38.52	376.40	0.00	2.00
Inter‐municipal mobility $\left(M\right)$	13.38	21.59	7.44	0.19
Population (in logs) $\left(P\right)$	8.57	12.44	3.87	0.26
Wealth (in logs) $\left(W\right)$	9.63	10.47	9.18	0.03
Immigration (in logs) $\left(\mathrm{IM}\right)$	6.33	10.80	0.00	0.39
Age $\left(A\right)$	42.14	58.44	32.89	0.10
Tourism (in logs) $\left(T\right)$	4.40	10.32	0.00	0.52
Spatial lag of number of infections ( WCinf)	44.55	235.88	1.56	1.19

*Source*: Open Data Portal of Madrid, INE, Statistical Institute of Madrid, Madrid City Council website, and own elaboration.

Table 3 reports the estimation results of our model by maximum likelihood because it has sound statistical properties in a spatial model for the whole period (first column) as well as every landmark date detailed above (remaining columns). In all cases, robust standard errors are used. Starting with the whole period, our findings tend to confirm several of the hypotheses: H1 (the role of mobility as a driver), H2 (a positive link between population and infections), H3 (a negative relationship between wealth and infections), H5 (the older the population the higher the number of infections), and H7 (the link between neighbouring municipalities). The remaining hypotheses (those related to immigration H4 and tourism H6) do not seem to be validated for the entire period.

**TABLE 3:** Number of COVID‐19 infections: Some explanatory factors

Dependent variable: ${\text{Cinf}}_{i}$	Whole period (06/03–21/06)	Before lockdown (06/03)	Start of lockdown (15/03)	Peak of the pandemic (29/03)	End of lockdown/de‐escalation (Phase 0) (27/04)	De‐escalation (Phase 0 is extended) (10/05)	De‐escalation (Phase 1) (25/05)	De‐escalation (Phase 2) (08/06)	End of state of alarm (21/06)
	Coeff. (*p* value)	Coeff. (*p* value)	Coeff. (*p* value)	Coeff. (*p* value)	Coeff. (*p* value)	Coeff. (*p* value)	Coeff. (*p* value)	Coeff. (*p* value)	Coeff. (*p* value)
α	−162.64 (0.191)	8.52 (0.649)	25.18 (0.814)	−188.45 (0.628)	−485.07*** (0.002)	−150.05*** (0.009)	−76.31** (0.019)	−39.99 (0.319)	21.09 (0.501)
$M_{i}$	3.60*** (0.002)	0.10** (0.029)	1.50*** (0.001)	11.39 (0.137)	0.65 (0.577)	0.68 (0.247)	0.55* (0.060)	0.46** (0.029)	0.45*** (0.008)
$P_{i}$	18.70*** (0.007)	1.34** (0.017)	12.25** (0.050)	50.86** (0.028)	13.11** (0.036)	8.54*** (0.010)	5.24*** (0.000)	4.86*** (0.008)	3.29** (0.023)
$W_{i}$	−34.01** (0.028)	−2.52 (0.245)	−30.16** (0.048)	−123.35** (0.021)	5.57 (0.726)	−2.79 (0.679)	−3.54 (0.308)	−7.23 (0.110)	−9.57** (0.018)
${\mathrm{IM}}_{i}$	2.00 (0.640)	−0.50 (0.206)	−1.08 (0.786)	12.51 (0.330)	5.74 (0.175)	−0.53 (0.777)	0.15 (0.888)	0.04 (0.968)	−0.19 (0.828)
$A_{i}$	6.57*** (0.000)	0.10 (0.106)	2.80*** (0.001)	19.66*** (0.000)	6.82*** (0.000)	2.50*** (0.000)	1.50*** (0.000)	1.56*** (0.000)	0.90*** (0.000)
$T_{i}$	1.43 (0.248)	0.25** (0.048)	3.06** (0.012)	1.54 (0.686)	0.13 (0.935)	0.32 (0.659)	0.02 (0.940)	−0.60 (0.215)	−0.01 (0.972)
$\sum_{j}w_{\mathrm{ij}}{\text{Cinf}}_{j}$	0.54*** (0.000)	−0.02 (0.936)	0.41 (0.135)	0.58*** (0.000)	0.64*** (0.000)	0.54*** (0.000)	0.38*** (0.007)	0.54*** (0.000)	0.65*** (0.000)
Pseudo‐*R* ^2^	0.778	0.235	0.512	0.747	0.801	0.726	0.698	0.668	0.655
Log‐likelihood	−996.06	−518.29	−988.81	−1228.45	−1000.40	−836.07	−723.19	−729.10	−584.50
Number observ.	199	199	199	199	199	199	199	199	199

*Note*: *Cinf*, number of infections; *α*, constant term; *M*, inter‐municipal mobility; *P*, population; *W*, wealth; *IM*, immigration; *A*, age; *T*, tourism; $w_{\mathrm{ij}}$, elements of the spatial‐weight matrix *W* between each pair of municipalities *i* and *j*.

*** Significant at 1%.

** Significant at 5%.

* Significant at 10%.

*Source*: Open Data Portal of Madrid, INE, Statistical Institute of Madrid, Madrid City Council website, and own elaboration.

Turning to H7 (the link between neighbouring municipalities), space matters when it comes to understanding factors behind the spread of the pandemic. The implied message is unambiguously stark: a multivariate analysis that does not consider the connection between nearby areas—that is, without including spatial spillovers—can be completely misleading. It is indeed exceptionally important when alternative scenarios are considered in terms of measures of social distancing.^8^

As for the remaining findings, in terms of H1 (mobility) it appears, by applying the estimated coefficient $\beta_{2}$, that a 10% increase in the share of people who move regularly would result in about 36 new infections (in a period of 14 days) and in a context of free movements, each additional carrier could propagate new contagions. Our finding, hence, confirms the importance of adopting mobility‐based restrictions.

Our work also settles the significance of population size (H2), and more importantly, the role played by age (H5); for every 1‐year increase in the median age of a municipality, the results indicate that there would be seven new infections, a finding likely connected to the period we were studying, in which testing was very limited and confined to hospitals. In a different scenario of mass screening for asymptomatic patients, the results would probably change.^9^ Finally, there is some evidence suggesting that poor municipalities are more likely to suffer from the pandemic (H3). This finding aligns with other studies concluding there is a more considerable risk of infection in low‐income communities (Hamidi & Hamidi, 2021). In any case, an increase of 10% in income would reduce the number of infected people by just over three units, so the effect is not particularly strong.

Given these general results, a crucial point mentioned above must be borne in mind: The results may mask significant changes over the period. Therefore, close attention has been needed to the rest of the columns in Table 3, after which we have drawn noteworthy conclusions about how leading factors in the spread of COVID‐19 change as different preemptive measures to contain the virus are imposed/relaxed.

The first date (6 March, Table 3) is the only one with a relatively low goodness‐of‐fit for our model (pseudo‐*R* ^2^ of 0.235). Indeed, only three variables turn out to be statistically significant (and positive), namely, population, mobility, and tourism, with no “geographical factor” at this time. The message conveyed by these results is meaningful: In the early stages, the distribution of infections has an apparently random character difficult to capture in a model (low pseudo‐*R* ^2^); yet, it is somewhat connected to factors related to the flow of people between areas. In other words, apart from the fact that everybody, every area, can be affected, chances are greater for tourist destinations because of their idiosyncrasies as crowded places with people in close proximity, especially if they are also featured by the mobility of their local citizens. This result is consistent with the evidence reported by Farzanegan et al. (2021). With a sample of more than 90 countries and data as of 30 April 2020, they show a higher exposure to COVID‐19 for countries with higher international tourism flows.

Considering the nationwide lockdown imposed on 15 March (Table 3), the main point is an incipient geographic pattern. The sign of the spatial lag of the dependent variable becomes positive and almost different from zero at the 10% level. This fact is surely related to the mobility variable, whose positive and significant coefficient increases. Additionally, income emerges as a COVID‐19 driver, as expected with a negative sign. Apart from tourism, which still retains its importance, age emerges as a significant factor of COVID‐19 propagation for the first time. Linking all these results, we would cautiously assert that a core–periphery pattern, common in other contexts, is identifiable, comprising a dense and well‐connected core made up of tourist municipalities with a high level of citizen mobility, together with a periphery made up of municipalities with lower income.^10^

As a rule of thumb, during the lockdown (Table 3, columns 4 and 5), the geographical pattern, showing the existence of spatial contagion between neighbouring municipalities, is getting stronger and becoming heavily significant (thus reinforcing H7). To be precise, the increase in the corresponding parameter is especially significant in the first part of the confinement period. We could state that the seed for the spread of the virus between nearby areas had been already planted, mainly through mobility, prior to the setting up of the lockdown, leaving its devastating effects afterwards.

As expected, because of mobility restrictions and social isolation with the lockdown, the mobility variable (the same is true for tourism) is not statistically significant either on 29 March (peak time) or 27 April (end of the lockdown). However, this does not mean they are blameless because their effects occur with a certain delay. The point is that once mobile citizens spread the virus between municipalities, it is difficult to stop its dissemination within those areas without high levels of traceability. Obviously, the problem is acute in highly populated areas with ageing populations (these variables are significant on both dates) and even more so in relatively poor areas (significant at the peak of the pandemic).

During the de‐escalation phase, on 10 May, when Phase 0 was extended for Madrid, the geographical pattern, while starting to lose importance, is still remarkable. Even after a prolonged period of limited mobility, and despite much lower overall pandemic figures, it is hard to clear initial hotspots.

Moving to the analysis of the start of Phase 1, on 25 May, to avoid repetition, we restrict our attention to the mobility variable; its coefficient becomes again positive and significant (at 10%; Table 3). This result conveys an explicit message: after a stay‐at‐home order, if you loosen restrictions too fast, it takes time, but mobility reappears as one of the dominant drivers of the pandemic. In effect, in Phase 2 of the de‐escalation (8 June), the role of mobility is getting stronger (significant at 5%), and probably because of that, the link between neighbouring municipalities (coefficient of the spatial lag of the dependent variable) increases again.

These observations are reinforced in relation to data pertaining to the latest date (21 June). At the same time, income again becomes negative and significant, which we could interpret as a revival of the core–periphery pattern associated, in part, with mobility, including to remote and comparatively poor municipalities that had been almost completely “free” of the virus at the beginning of the wave. Accordingly, loosening of restrictions in the de‐escalation phases seems to be paving the way for new peaks in infections caused, largely, by people’s mobility. Unfortunately, this finding has been confirmed by later pandemic waves.

## 5 DISCUSSION AND CONCLUSION

The main aim of this article has been to uncover factors that have driven the expansion of the COVID‐19 pandemic, taking the region of Madrid as a case study. A spatial ecological model was proposed and subsequently estimated for different key dates of the first wave. The importance of driving factors differs significantly between the various phases of the pandemic depending on public health measures taken to curb community transmission. Initially, the spread of the disease was mainly focused on highly populated municipalities characterised by high mobility and affluence, including from tourism. Thereafter, a spatial contagion process showed up in the time it took for the pandemic to expand out. Afterwards, mobility‐based restrictions started having significant effects on the propagation of the pandemic, which slowed down, and led to a reduction of cross‐municipal spillovers. We think that mobility between municipalities in metropolitan areas contributed to the emergence of geographical clusters of municipalities at high risk of infection, which remained active due to difficulties in reducing disease incidence within their borders. In addition, areas with the largest elderly populations were the most vulnerable in the first stage of the pandemic, where the fight against the pandemic took the longest and required the most effort. There is some evidence to support the idea that municipalities with lower disposable income were also and remain more prone to pandemic surges.

Results following the lockdown period provide lessons for the future. On one hand, in line with an idea put forward by Alfano and Ercolano (2020), the effectiveness of this kind of measures was maintained for some time even after their lifting. However, as restrictions were progressively loosened during the de‐escalation phase, inter‐municipal mobility drove contagion and reinforced geographical patterns involving age, deprivation, and so on. Therefore, policymakers should remain vigilant about people’s mobility, even, or even especially, in de‐escalation phases.

To test this point, we conducted a straightforward counterfactual analysis. We used the model results for the whole period to predict what the number of contagions would have been without lockdown; put differently, under the assumption that the level of mobility had consistently remained at its values before the lockdown. We are aware of the simplicity of our approach but think it can provide a view of the importance of non‐pharmaceutical interventions and their potential order of magnitude. The result is noteworthy. The average number of infections for a period of 14 days was actually 7,665; in our simulation, it reached a value of 20,675. In other words, the number of infections would have been, ceteris paribus, almost 170% greater.

Accordingly, one of the critical lessons to be drawn from this case study is that restrictions are going to be, to a greater or lesser extent, part of life until vaccination levels reach high levels (herd immunity),^11^ or alternatively, COVID‐19 infections have sound treatments. Either way, we have established that once a geographical pattern is discernible, public action is critical to contain the propagation of the disease (see Karnon, 2020).

On the basis of the foregoing, we argue that, instead of complete confinement, specific “local/partial lockdowns” mobility restrictions should be implemented because they would be less costly both socially and economically.^12^ We suggest that efforts should be concentrated in the most populated areas with high levels of population mobility and a strong tourism sector. In such areas, there should be random mass screening and teams of well‐trained “trackers” (familiar, for instance, with the area and its neighbourhoods) ready to act to enforce lockdowns and distancing. Done efficiently, local lockdowns might be enough to bring the spread of the virus under control.

Finally, it is important to design and apply tools that enable the development of a spatial contagion index that serves as a reference for appraising when the speed of the spread of the virus between nearby areas exceeds an already established benchmark. Such a tool could help policymakers gauge the best time to implement pre‐designed measures based, for the time being, on non‐pharmaceutical interventions. If data were available, it would also be interesting to re‐run the study using, for instance, neighbourhood or even household information. That being so, in the proposed model, we could include auxiliary variables, such as housing quality, or information about whether or not family members are engaged in activities considered essential during the pandemic and/or in “risky” occupations. Finally, a third extension would involve another potential replication for other regions with characteristics different from those in Madrid. In that way, we could test the external validity of results. In other words, we would have a more precise picture of how, for instance, the effectiveness of social/physical distancing interventions, as well as the drivers of COVID‐19 spread, may depend on the sampling location.

## ACKNOWLEDGEMENT

No funding was received for this paper.

## CONFLICT OF INTEREST

The authors declare that they have no conflicts of interest.

## ETHICS APPROVAL STATEMENT

Ethics approval was not required for this study.

## Supporting information


**Data S1.** Supporting information

**Data S2.** Supporting information

## Data Availability

The data that support the findings of this study are openly available in Figshare at https://figshare.com/articles/dataset/Data_COVID_pandemic_in_Madrid/14306861.

## ENDNOTES

1 There is inevitably a trade‐off here, because the higher the level of disaggregation, the lower the availability of data. Given this problem, we chose municipal data for two reasons: on the one hand, to try to minimise the so‐called ecological fallacy (drawing conclusions about small units from empirical results at higher levels of aggregation); on the other hand, because it is the maximum level of disaggregation for which the quality of the data is high.

2 To assess the expansion of the disease, we consider the number of cases is more suitable than the number of deaths.

3 Likewise, Stojkoski et al. (2020) propose a model with six very similar categories: Healthcare Infrastructure, National Health Statistics, Economic Performance, Societal Characteristics, Demographic Structure, and Natural Environment.

4 It is quite clear, however, that this is not the sole factor. As indicated by Boterman (2020) for the case of the Netherlands, the geography of the pandemic is more complex. We try to capture its complexity in our proposed model.

5 As indicated by a referee, the inclusion of immigration could cause endogeneity and/or at least correlation problems among the predictors of the model. In any case, when the immigration variable is excluded, the results obtained are basically the same.

6 Another variable we thought about was the size house or, as it is commonly called, crowding ratio, defined as the average number of people per principal housing. It is straightforward that the contagion probabilities between people living in the same house are the highest. However, the reason why we ultimately did not include it is that we found multicollinearity problems with *Age*.

7 Another option, not addressed in this paper, would be to use the connectivity between municipalities rather than the physical distance to compute the distance matrix (see, for example, Iacus et al., 2020).

8 This conclusion also underlies mathematical models of infectious diseases. In this field, it is worth mentioning the lack of ability of traditional compartmental epidemiological models, compared with network models, to adequately explain the speed of spread of COVID‐19 disease between individuals (Komaroca & Wodarz, 2020). This is another way of capturing, from a different perspective, the same idea: the need to include spillover effects between neighbouring areas when modelling.

9 Throughout the sample period, not only Madrid but also Spanish strategy was simply testing symptomatic patients. This strategy may be suboptimal, as indicated by Padula (2020), but at that time, there was a noticeable problem of lack of resources.

10 As noted in several studies such as those by Ascani et al. (2020), Ramírez‐Aldana et al. (2020), and Amdaoud et al. (2021), largest cities, especially those close to international major airports, are usually the origin of the COVID‐19 outbreak, creating a major spatial hotspot from which the virus propagates to other peripheral areas.

11 There is much debate as to when herd immunity would be achieved. Although vaccination is not the only relevant factor, it seems that the target figure in this respect is around 70%.

12 Although beyond the scope of this paper, a quite important point related to this issue has to do with the ability of municipality governments, typically under‐resourced and subject to jurisdictional constraints, to deal with innovative governance initiatives, either alone or in networked alliances (McGuirk et al., 2021).
