# Supplementary material for: Modelling changing patterns in the COVID‐19 geographical distribution: Madrid’s case
Source: Geographical Research. 2021 Nov 9;60(2):218–31. doi: 10.1111/1745-5871.12521 (PMC8652501; doi:10.1111/1745-5871.12521)
Supplement: Supplementary file 1 — Data S1. Supporting information [file GEOR-60-218-s002.docx]

**Online Appendix 1**

To compute the inter-municipal mobility variable, we exploited data coming from *Studies on mobility based on mobile phone* published by Spanish National Statistical Institute (INE). These data correspond to ‘mobility areas’, which not always coincide with municipalities. As a way of example, data refer to inter-census areas mobility (for the municipality of Madrid), to inter-district mobility (for other municipalities), and so on. To be precise, here we faced two important problems.

1. On the one hand, data are only available for the period from 16 March to 20 June 2020, whereas our period started on March 6. In other words, we needed data for the pre-lockdown period. Consequently, we took the data from a *Pilot Study of Mobility* previously published by INE. Specifically, these data were collected on November 18, 2019.
2. In line with the previous comments, data are offered at a different level of disaggregation, which corresponds to what is called ‘mobility areas’. These mobility areas only in some cases match with our unit of analysis (municipalities). Then, to have a complete sample, we proceeded as follows:

a) Needless to say, if the mobility areas coincided with the municipality, we directly took the corresponding data.

b) For some relatively large municipalities the source provides data at the district level. In these cases, we simply calculated the mean values between districts.

c) For the case of the municipality of Madrid, although we are already using COVID data at district level due to its size, original mobility data are offered at the census area level. This being so, we grouped all census data that corresponded to each of the 21 districts conforming the municipality, and calculated mean values between them.

d) In some cases, data are offered for two municipalities jointly, let us say “*municipality A and B*”. In these occasions, implying always neighbouring municipalities, we assigned the same data of mobility to both of them.

e) In other cases, data are given for a group of municipalities, indicating only the name of one of them. To be precise, data are offered for “*municipality X and others*”. This situation occurred in 14 cases. Here we, first, identified those municipalities that were included under the umbrella of “*others*” by simply checking those that were not named in the data source; they were thus included in “*others*”. Second, we ‘guessed’ the group of “*others*” in which they were masked by identifying the “*municipality X*” that was closer to each; we rely on this procedure because, as mentioned above, the groups are formed on a geographic basis. Finally, we gave these municipalities the same value as the one collected for the “*municipality X*”.
