# Supplementary material for: Modelling changing patterns in the COVID‐19 geographical distribution: Madrid’s case
Source: Geographical Research. 2021 Nov 9;60(2):218–31. doi: 10.1111/1745-5871.12521 (PMC8652501; doi:10.1111/1745-5871.12521)
Supplement: Supplementary file 2 — Data S2. Supporting information [file GEOR-60-218-s001.docx]

**Online Appendix 2**

In this Appendix we aim at confirming the presence of spatial dependence in the number of contagions at municipal level. To do so, we compute, for the different dates studied, the most widely used and best known tests for spatial dependence: namely, Moran’s I and Geary’s C statistics.

Moran’s I ($MI$) statistic reads as follows:

$MI(t)=\frac{N}{\sum_{i=1}^{N} \sum_{j=1}^{N} w_{ij}}\frac{\sum_{i=1}^{N} \sum_{j=1}^{N} w_{ij}\left[ C{inf}_{i}\left( t \right)-Cinf(t) \right]\left[ {Cinf}_{j}(t)-Cinf(t) \right]}{\sum_{i=1}^{N} \left[ {Cinf}_{i}(t)-Cinf(t) \right]^{2}}$ $for i\neq j$ (A.1)

where ${Cinf}_{i(j)}$ denotes the number of infections in municipality $i (j)$, $Cinf$ is the mean number of infections in the region of Madrid, $w_{ij}$is an element of the distance matrix $W$ between each pair of municipalities, $\sum_{i=1}^{N} \sum_{j=1}^{N} w_{ij}$is a standardization factor that corresponds to the sum of all the weights, $N$ is the total number of municipalities, and $t$ denotes time. Under the null hypothesis of no spatial autocorrelation, the expected value of $MI$ is given by $E\left( MI \right)=-1/(N-1)$, being inference based on Z-values computed as $Z_{MI}=\frac{\left[ MI-E\left( MI \right) \right]}{\sigma\left( MI \right)}.$ A significant positive value of standardized Moran’s I ($Z_{MI}$-value) indicates positive spatial dependence (autocorrelation), while a significant but negative value corresponds to a pattern of spatial association between opposite values.

Geary’s C ($GC$) statistic is given by:

$GC(t)=\frac{N}{2\sum_{i=1}^{n} \sum_{j=1}^{n} w_{ij}}\frac{\sum_{i=1}^{n} \sum_{j=1}^{n} w_{ij}\left[ {Cinf}_{i}(t)-{Cinf}_{j}(t) \right]}{\sum_{i=1}^{n} \left[ {Cinf}_{i}(t)-Cinf(t) \right]^{2}}$ $for i\neq j$ (A.2)

denoting each letter the same as before. Under the null hypothesis of no spatial autocorrelation, the expected value of *GC* equals to 1, while Z-values are computed as $Z_{GC}=\frac{\left[ GC-1 \right]}{\sigma\left( GC \right)}$. However, contrary to Moran’s I, a positive (negative) and significant $Z_{GC}$-value shows negative (positive) spatial correlation.

The results obtained for both statistics are reported in Table A.1. As explained in the main text of the paper, the square of the inverse of the standardized distance is employed as the distance matrix. In any case, once again these results are quite robust, i.e., they do not change significantly when alternative distance matrices are used, such as the contiguity matrix, the inverse of the distance, or matrices considering different cut-offs.

Our analysis clearly reveals that there is positive spatial dependence in the number of infections whatever the date you are assessing. However, it is also important to note that there are some ups and downs all over the period, being the results obtained by the two statistics quite in sync. In any case, spatial dependence is one of the main features of the distribution, so it is important to stress that any analysis overlooking these spatial links could be misleading, if not plainly wrong. Accordingly, the spatial lag of the dependent variable is included in the proposed model trying to unveil some of the main factors behind the evolution of the number of infections.

Table A.1. *Spatial dependence*

| Dates | Moran’s I statistic | | | Geary’s C statistic | | |
| --- | --- | --- | --- | --- | --- | --- |
|  | $\boldsymbol{MI}$ | Z- value | p-value | $\boldsymbol{GC}$ | Z- value | p-value |
| **Whole period**  (06/03-21/06) | 0.579 | 21.07 | 0.000 | 0.511 | -12.65 | 0.000 |
| **Before lockdown**  (06/03) | 0.138 | 5.80 | 0.000 | 0.800 | -2.84 | 0.002 |
| **Start of lockdown**  (15/03) | 0.366 | 13.83 | 0.000 | 0.654 | -6.82 | 0.000 |
| **Peak of the pandemic**  (29/03) | 0.562 | 20.50 | 0.000 | 0.528 | -11.95 | 0.000 |
| **End of lockdown/De-escalation (Phase 0)**  (27/04) | 0.623 | 22.63 | 0.000 | 0.478 | -13.85 | 0.000 |
| De-escalation (Phase 0 is extended)  (10/05) | 0.560 | 20.44 | 0.000 | 0.531 | -11.80 | 0.000 |
| **De-escalation (Phase 1)**  (25/05) | 0.513 | 18.76 | 0.000 | 0.569 | -10.81 | 0.000 |
| **De-escalation (Phase 2)**  (08/06) | 0.496 | 18.29 | 0.000 | 0.592 | -9.40 | 0.000 |
| **End of State of Alarm**  (21/06) | 0.489 | 18.27 | 0.000 | 0.594 | -8.41 | 0.000 |
